# Inverse correlation between Tax and CD25 expressions in HTLV-1 infected CD4 T-cells in vivo

**DOI:** 10.1186/1742-4690-8-S1-A14

**Published:** 2011-06-06

**Authors:** Kenta Tezuka, Runze Xun, Mami Tei, Takaharu Ueno, Masakazu Tanaka, Norihiro Takenouchi, Jun-ichi Fujisawa

**Affiliations:** 1 Dept. Microbiology, Kansai Medical University, Moriguchi, Osaka, 570-8506, Japan

## 

Tax has pleiotropic actions that induce proliferation and inhibit apoptosis of T-cells and thus is considered to play a critical role in leukemogenesis. However, Tax expression is frequently lost in fresh adult T-cell leukemia (ATL) cells by genetic changes or epigenetic modifications of proviral genome. To clarify the significance of tax gene in leukemogenesis, we analyzed the expression of tax gene in the HTLV-1 infected human CD4+ T-lymphocytes in humanized mouse system.

NOG-SCID mouse were transplanted with human hematopoietic stem cells and infected with HTLV-1 in vivo by peritoneal injection of MT-2 cells after 4 months of transplantation. HTLV-1 infection induced the rapid proliferation of CD4+ T-cells, irrespective of the expression of CD25, and resulted in the severe splenomegaly with CD25+ CD4+ T-cells. Tax gene expression was low in the isolated splenocytes but greatly increased by the ex vivo culture for 24 hours as seen in infected peripheral blood T-cells from HTLV-1 carrier. As the gene expression of CD25 was also activated in proportion to the tax induction, function of Tax was indicated to be responsible for the CD25 expression.

When CD4+ T-cells from HTLV-1 infected splenocytes were separated into CD25+ and CD25- cells, however, tax expression was observed mainly in CD25- CD4+ T-cells. These results indicate that the expression of CD25 may not necessarily involve the Tax function in vivo. We will discuss the function of Tax in CD25- CD4+ T-cells and a significance of CD25 expression without the action of Tax in vivo.

